# Pathotyping Systems and Pathotypes of *Plasmodiophora brassicae*—Navigating toward the Optimal Classification

**DOI:** 10.3390/pathogens13040313

**Published:** 2024-04-11

**Authors:** Nazanin Zamani-Noor, Małgorzata Jędryczka

**Affiliations:** 1 Institute for Plant Protection in Field Crops and Grassland, Julius Kühn-Institute (JKI), D-38104 Braunschweig, Germany; 2 Pathogen Genetics and Plant Resistance Team, Institute of Plant Genetics, Polish Academy of Sciences, 60-479 Poznań, Poland

**Keywords:** clubroot, physiological specialization, virulence and pathogenicity, genetic diversity, race differentiation, pathogen variability, disease management, host–pathogen interaction, Brassicaceae, crucifers

## Abstract

*Plasmodiophora brassicae* Woronin, an obligate biotrophic soil-borne pathogen, poses a significant threat to cruciferous crops worldwide by causing the devastating disease known as clubroot. Pathogenic variability in *P. brassicae* populations has been recognized since the 1930s based on its interactions with Brassica species. Over time, numerous sets of differential hosts have been developed and used worldwide to explore the pathogenic variability within *P. brassicae* populations. These sets encompass a range of systems, including the Williams system, the European Clubroot Differential set (ECD), the *Brassica napus* set, the Japanese Clubroot Differential Set, the Canadian Clubroot Differential Set (CCS), the Korean Clubroot Differential Set, and the Chinese Sinitic Clubroot Differential set (SCD). However, all existing systems possess both advantages as well as limitations regarding the detection of pathotypes from various Brassica species and their corresponding virulence pattern on Brassica genotypes. This comprehensive review aims to compare the main differential systems utilized in classifying *P. brassicae* pathotypes worldwide. Their strengths, limitations, and implications are evaluated, thereby enhancing our understanding of pathogenic variability.

## 1. Introduction

*Plasmodiophora brassicae* Woronin, an obligate biotrophic soil-borne pathogen, poses a substantial menace to cruciferous crops worldwide by causing a devastating disease known as clubroot. The pathogen presents a significant threat to agriculturally important plants within the Brassicaceae family, including oilseed rape, cabbage, broccoli, cauliflower, and various mustard species [1,2,3,4,5]. First identified in the late 19th century [6], the pathogen has since accumulated attention for its ability to induce the formation of distinctive club-shaped galls on the roots of infected plants, leading to reduced nutrient uptake, stunted growth, and ultimately compromising crop yield [7,8,9,10,11].

The life cycle of *P. brassicae* begins with the activation of durable resting spores in the soil, germinating under favorable conditions and giving rise to motile zoospores [9,11,12,13]. These zoospores move toward susceptible plant roots, initiating infection. Within the root tissues, the pathogen undergoes a series of developmental stages, forming primary zoosporangia. These structures release secondary zoospores, facilitating the spread of the infection within the roots [14]. A distinctive life cycle feature is the induction of galls on the roots, creating a conducive environment for the pathogen’s sustenance. As the galls mature, they release resting spores into the soil, completing the cycle. The longevity and resilience of these resting spores contribute to the persistence of *P. brassicae*, presenting an ongoing challenge in managing clubroot disease [15].

A critical aspect of *P. brassicae* lies in the existence of pathogenic variability [16], referred to as a pathotype [8]. According to the definition a pathotype, sometimes also called a virulence phenotype, is any group of organisms of the same species that shows the same pathogenicity on a specified host. Pathotypes represent distinct variants of the pathogen that exhibit varying levels of infectivity on specific host cultivars, revealing a complex interplay between host resistance and pathogen diversity. Pathotypes highlight the ability of *P. brassicae* to diversify and adapt to given conditions [17,18]. Due to genetic variability, numerous isolates of *P. brassicae* with different pathogenicity can be found worldwide [19,20,21,22,23,24,25,26]. 

Developing clubroot-resistant cultivars is a predominant and sustainable strategy in clubroot management. To date, several clubroot-resistant cultivars of Chinese cabbage and oilseed rape/canola have been released across Europe, China, Japan, Korea, and Canada. However, the presence of pathotype-specific clubroot resistance genes, coupled with pathotype variability, contributes to the eventual loss of resistance in these cultivars within 3 to 4 years [19,27,28]. Therefore, classifying different pathotypes is essential for understanding the subtle interactions between the pathogen and its cruciferous hosts.

The molecular detection of *P. brassicae* in plants and soil samples is feasible [24,29,30,31,32] but it does not detect the pathotypes. The pathotypes of *P. brassicae* are differentiated based on their observable characteristics in bioassays using specific host differential sets. Host reactions are visually assessed within these assays by monitoring root gall development. To date, multiple sets of differential hosts, such as the Williams system, the European Clubroot Differential set, the *Brassica napus* set created by Somé et al., the Canadian Clubroot Differential set, and the Chinese Sinitic Clubroot Differential set, have been developed and utilized worldwide to investigate the pathogenic variability in *P. brassicae* populations [33,34,35,36,37]. The expansion of these differential host series makes it challenging, if not impossible, to interpret data from a global perspective [38].

In the present review, our objective is to compare the main differential systems for classifying *P. brassicae* pathotypes worldwide. We evaluate their strengths, limitations, and implications, thereby enhancing comprehension of the subtle variations in pathogenic variability.

### 1.1. American Differential Set

The differential set introduced by Williams in 1966 remains one of the primary systems worldwide for classifying *P. brassicae* pathotypes [33]. This system relies upon two distinct hosts of *Brassica napobrassica* (cv. Laurentian and cv. Wilhelmsburger) and two hosts of *B. oleracea* (cv. Badger shipper and cv. Jersey Queen), thereby facilitating the differentiation of 16 pathotypes or races (Table 1). The study involved 36 isolates of *P. brassicae*, primarily collected from cabbage *B. oleracea* (16 isolates) and cauliflower *B. oleracea* var. *botrytis* (6 isolates). Additionally, isolates were sourced from rutabaga *B. napus* var. napobrassica (4 isolates), turnip *B. rapa* subsp. *rapa* (4), kohlrabi *B. oleracea* var. *gongylodes* (2), Chinese cabbage *B. rapa* (2; the specific subspecies used was not specified), and oilseed rape *B. napus* (1). Moreover, one isolate of *P. brassicae* was obtained from candytuft *Iberis sempervirens*, which also belongs to the Brassicaceae family. In total, two-thirds of the tested isolates (24 out of 36) originated from various forms of cabbages belonging to *B. oleracea*, while only two isolates were derived from oilseed rape *B. napus*.

The tested isolates were sourced from various regions, including Australia (5), Canada (3), Czechoslovakia (1, presently located in the Czech Republic), England (2), Finland (2), Germany (8), Japan (3), New Zealand (4), Norway (3), USA (4), and Russia (1). This global collection represented significant diversity in isolates and encompassed geographically distant locations. All tested isolates fell into 9 categories, prompting the investigator to assign variant numbers 1–9 out of a possible 16 combinations. In the discussion, it was suggested by the author that some of the races found by other researchers would undoubtedly add to the nine races identified in his study. Despite the absence of known resistance genes, different combinations of plant reactions were classified as races, rather than pathotypes.

The decontaminated soil was evenly mixed with spore suspension in distilled water to obtain a concentration of 10^8^ *P. brassicae* spores/cc of soil. Seven-day-old seedlings of the differential hosts (Table 1) were transplanted from vermiculite to the infected soil, grown for 35 days, and then delicately pulled out and rated for disease symptoms. In the William system, the evaluation scale ranged from zero (indicating no galls) to three (signifying severe clubs on primary and secondary roots). The number of plants classified in class 0 was multiplied by 0, those in class 1 by 10, those in class 2 by 60, and those in class 3 by 100. The disease index (DI) was then determined by dividing the summed value by the total number of tested plants. If the DI fell below 90, the test was repeated to ensure that the observed resistance stemmed from the plant genotype rather than the low viability of spores. To attain dependable outcomes, Williams [3] recommended using high spore loads of a pure race. Additionally, his study revealed intermediate scores on certain host differentials, which were attributed to mixtures of races.

The Williams’ differential set comprises just two rutabagas and two cabbages. While this system is straightforward and involves only a small number of hosts, thereby reducing greenhouse space requirements for pathotype assessments, it was primarily designed to distinguish strains of *P. brassicae* from cabbage and rutabaga. Consequently, a pathotype designation based on the Williams’ system may not comprehensively reflect virulence patterns across all Brassica species. For example, in a study conducted by Kim et al. [39], pathotype 4, as per the Williams’ system and widely distributed in China, Japan, and Korea, exhibited varying disease severities in Chinese cabbage. This observation underscores the potential limitations of the Williams’ system for accurate pathotype classification and effective clubroot resistance breeding in the Asian context. An additional challenge linked to the utilization of Williams’ differential set is the presence of inconsistent host reactions, often falling between resistant and susceptible categories [19,20]. In certain instances, this inconsistency stems from heterogeneity within the differential host genotypes, which typically comprise older open-pollinated cultivars. Such intermediate and fluctuating reactions can complicate the pathotype classification of specific *P. brassicae* populations or potentially result in divergent outcomes across different countries.

### 1.2. European Clubroot Differential Set

The European Clubroot Differential or ECD set is the second most widely employed system worldwide for analyzing *P. brassicae* populations (Table 2). This differential set comprises 15 distinct plant species categorized into 3 genomic subsets. The initial set of five hosts corresponds to the *B. rapa* genotype, the second group involves the *B. napus* genotype, and the third subset encompasses hosts derived from the *B. oleracea* genotype. Within the *B. rapa* hosts, combinations of two to three monogenic resistances (A, B, and C) are present, while *B. napus* and *B. oleracea* are composed of a number of genes of small effect, contributing forms of field or polygenic resistance [1,40].

In this system, each host receives a specific score in the event of an infection (Table 2). A numerical code, comprising three numbers representing the observed susceptibility of hosts within each of the three Brassica species, was then assigned to each *P. brassicae* isolate by adding the denary values of hosts within a species group showing a susceptible reaction with a disease severity index equal to or greater than 33 percent. Pathotype designations are systematically arranged in triplets and each genotype is counted independently. For example, ECD code 16/14/3 would represent a *P. brassicae* population that caused a susceptible reaction on *B. rapa* ECD 05, *B. napus* ECD 07, ECD 08, and ECD 09, as well as *B. oleracea* ECD 11 and ECD 12 (Table 2).

The ECD set theoretically has the capacity to identify 455 distinct pathotypes and has played an important role in resolving issues regarding the potential existence and importance or otherwise of physiological races or pathotypes of *P. brassicae*.

In the study conducted by Crute et al. [41], a comprehensive analysis was performed on 240 datasets obtained globally from ECD tests. Their findings indicated that the resistance to clubroot in *B. rapa* and *B. napus* was race-specific, while the resistance observed in the ECD hosts of *B. oleracea* appeared non-differential. However, the prevalence of *P. brassicae* populations capable of overcoming resistance in *B. rapa* hosts is significantly lower than isolates that can overcome resistance in *B. napus* and *B. oleracea* [24,26,28]. Interestingly, the observed pathogenicity towards resistant *B. rapa* hosts appears to be correlated with that exhibited toward resistant *B. napus* hosts. In contrast, resistant *B. oleracea* hosts tend to exhibit severe infections with *P. brassicae* isolates that lacked virulence on most resistant *B. rapa* and *B. napus* hosts [1,41]. This deviation in pathogenicity suggests the existence of distinct mechanisms for clubroot resistance that necessitate further exploration and understanding.

The ECD set has a sophisticated system of nomenclature, which has not been understood or accepted widely by farmers, breeders, or extension personnel, despite its ability to assist clubroot researchers in determining the resistance or susceptibility of differentials based on the ECD classification of each pathotype. Moreover, the large number of hosts necessitates a significant amount of greenhouse space as well as materials when characterizing *P. brassicae* populations.

### 1.3. French Differential Set

In France, variation in virulence was investigated across 20 field collections of *P. brassicae*. In total, 7 of the 10 Brassica lines subjected to testing displayed distinct reactions upon inoculation. Notably, two oilseed rape cultivars exhibited previously unreported differential responses. It was observed that some of the differential lines previously used to classify *P. brassicae* pathotypes were susceptible to all collections, suggesting potential divergence in pathogen populations between France and other reported regions. Consequently, the Somé differential set was developed, comprising three genotypes of *B. napus* (ECD 06, ECD 10, and the spring-oilseed rape cultivar Brutor). In this system, the clubroot disease index varied from 0 (no galls) to 100 and a cut-off point of 25% was used to classify reactions as virulent or avirulent. Furthermore, in their study, Somé et al. [35] used a single-spore isolate, indicating the use of only one spore for host inoculation. The outcomes provided insights into a genetically uniform pathotype. This method demands a substantial investment of time and effort, given that single-spore inoculation achieves success in only 10% of cases. The French Differential Set developed by Somé is predominantly used in Europe and can distinguish eight pathotypes (Table 3).

Similar to the differential set developed by Williams [33], some limitations have been identified within the differential set proposed by Somé et al. [35]. While this set comprises only three *B. napus* hosts that exhibit distinct reactions to the pathogen, its ability to differentiate pathotypes is relatively limited. Furthermore, this set exhibits even less capability for *P. brassicae* populations that have overcome the resistance in currently available clubroot-resistant oilseed rape cultivars. For instance, numerous *P. brassicae* populations have demonstrated moderate to high levels of virulence against the clubroot-resistant *B. napus* cv. Mendel [38,42,43,44]. These novel isolates have been provisionally designated as P1 (+), P2 (+), or P3 (+) as they correspond to P1, P2, or P3 on the differentials developed by Somé et al. [35] but unlike the original P1, P2, or P3 isolates, they exhibit high virulence against cv. Mendel.

### 1.4. Japanese Clubroot Differential Set

Due to the limitations of previous race differentiation systems, such as the Williams and ECD sets in distinguishing between pathogenic and nonpathogenic populations on clubroot-resistant cultivars of Chinese cabbage (*Brassica rapa* ssp. *pekinensis*) developed in Japan, Kuginuki et al. [19] introduced an innovative race-differential system. They aimed to elucidate the genetic diversity in the pathogenicity of *P. brassicae* in Japan using Japanese clubroot-resistant F1 hybrid cultivars and lines of *B. rapa* [19]. This system incorporated five clubroot-resistant F1 cultivars of Chinese cabbage (CR Kanko, CR Kukai 65, CR Ryutoku, CR Utage 70, and CR W-1116) along with a susceptible F1 cultivar of Chinese cabbage (Muso) as hosts. The incorporation of these cultivars enabled the identification of clear and distinguishable resistant or susceptible responses to *P. brassicae* populations from Japan. Utilizing this system, 36 *P. brassicae* field populations from Japan were successfully classified into four groups or pathotypes.

However, Osaki et al. [45] modified the classification system proposed by Kuginuki et al. [19] by using six clubroot-resistant *B. rapa* and a susceptible Chinese cabbage cultivar (cv. Nozaki Nigo) as differential hosts. Four pathotypes (A–D) were identified after inoculation of seven cultivars of Chinese cabbage with 17 *P. brassicae* populations from cruciferous crops (Table 4).

Lastly, yet importantly, the Chinese cabbage cultivar Utage 70 possesses a distinct and important genetic background, as described by Kuginuki et al. [19], and plays a significant role in the pathotype classification system. However, this specific cultivar is not commercially available and cannot be procured anymore. Consequently, Hatakeyama et al. [46] have proposed a modified differential system, wherein the Chinese cabbage cultivar Super CR Hiroki is a substitute for CR Utage 70.

### 1.5. Canadian Clubroot Differential Set

The observed significant variability in the virulence of *P. brassicae* across all differential sets [33,34,35], as well as the increasing *P. brassicae* populations capable of overcoming the resistance of resistant canola cultivars, indicates that these differentials did not fully capture the range of pathogenic diversity present in *P. brassicae* populations from Canadian canola [21]. Consequently, Strelkov et al. [36] developed a new classification system called the Canadian Clubroot Differential, or CCD set, which included the differentials of Williams [33] and Somé et al. [35], eight hosts of the ECD Set [34], and the clubroot-resistant *B. napus* cultivar Mendel [47], the open-pollinated canola cv. Westar, and the clubroot-resistant hybrid canola cv. 45H29. *P. brassicae* isolates were inoculated onto each host within the CCD set and the disease development was evaluated six weeks post-inoculation. In this system, the clubroot disease severity index varied from 0 to 100 and a cut-off point of 50% was used to classify reactions as susceptible or resistant. Pathotypes were outlined by observing host reactions, characterized by distinct virulence patterns that differentiate individual pathotypes. Populations of *P. brassicae* that produced a unique virulence pattern on the CCD set were assigned a letter (A, B, C, etc.) to distinguish between different pathotypes [21]. Since the CCD set includes all of the differentials of Williams [33] and Somé et al. [35], each population also was assigned a pathotype classification based on each of those systems [21].

A proposed draft for the classification of *P. brassicae* pathotypes on the CCD set is presented in Table 5, wherein 106 isolates, which were collected in Alberta/Canada, were classified into 17 unique pathotypes. However, the CCD set has a greater differentiating capacity [36].

Incorporating all differential hosts from Williams [33] and Somé et al. [35] into the CCD set allowed for determining pathotype designations based on these systems, facilitating direct comparisons. Each unique virulence pattern observed on the hosts within the CCD set was considered a distinct pathotype of *P. brassicae* and was denoted by a single uppercase letter for identification purposes. The authors mentioned that while several alternative pathotype nomenclature systems were evaluated, this set was chosen for its simplicity and ease of use in extension. Letters were preferred over numbers to prevent confusion with the numbering systems of Williams or Somé et al. If the total number of pathotypes identified using the CCD set surpasses the number of letters in the English alphabet, additional pathotypes could be designated using Greek letters or combinations thereof.

The inclusion of two clubroot-resistant *B. napus* cultivars, Mendel and 45H29, in the CCD set facilitated the identification of *P. brassicae* populations capable of overcoming clubroot resistance in the existing rapeseed cultivars resistant to clubroot. Segregation ratios from crosses with cv. Mendel demonstrated the involvement of at least one dominant and two recessive genes in this cultivar [47]. Moreover, the authors asserted that the spring oilseed rape cv. ‘Brutor’ was included in the CCD set because it was one of the differentials utilized by Somé et al. [35]. Nevertheless, ‘Brutor’ exhibited susceptibility to all of the tested populations of *P. brassicae*, thus failing to contribute to differentiation capacity. While excluding it from future studies could reduce the total number of differential hosts, it would be impossible to assign pathotype designations according to Somé et al. [35]. Similarly, the old canola cultivar ‘Westar’ showed susceptibility to all tested pathogen populations and given that ECD 05 already serves as a susceptible check, its removal from the CCD set appears justified. Eliminating ‘Brutor’ and ‘Westar’ would reduce the total number of CCD differentials to 11, thereby simplifying assessments of physiological specialization in the clubroot pathogen and conserving time and space.

### 1.6. Korean Clubroot Differential Set

The research team from Korea proposed the use of four distinct hosts of Chinese cabbage as an effective screening system for identifying resistant cultivars of *B. rapa* [39]. They specifically focused on Chinese cabbage as the primary source of *P. brassicae* isolates, using 11 isolates derived from Chinese cabbage and one isolate from cabbage (*B. oleracea*). These isolates were categorized into five pathotypes using the Williams differential set [33]. Subsequently, these isolates were inoculated onto clubroot-resistant cultivars of Chinese cabbage, revealing differential responses among isolates previously classified as the same pathotype by Williams. From an initial pool of 22 Chinese cabbage cultivars from Korea, China, and Japan, the authors identified four cultivars that seemed suitable for resistance breeding in clubroot-resistant Chinese cabbage (Table 6). Later on, populations of *P. brassicae* were inoculated into each host and the severity of club development on each plant was assessed using a scale ranging from 0 to 4, as outlined by Kuginuki et al. [19]. Hosts exhibiting disease severity (DS) ratings of ≤1 were classified as ‘resistant,’ whereas those with DS ratings of ≥2 were labeled as ‘susceptible’ (Table 6).

The investigation revealed that all field isolates examined in this study were classified into the four groups outlined in Table 6. However, the study encountered limitations due to the restricted number of *P. brassicae* isolates, with exclusive use of *B. rapa* as hosts. The ‘Korean’ system proposed by Kim et al. [39] is primarily adapted for Chinese cabbage, yet its applicability for diversifying isolates originating from winter oilseed rape or spring canola is constrained by the insufficiency of host differentials. To validate the efficacy of this system for Chinese cabbage breeding, it is imperative to assess a larger sample size of isolates from Asia, as well as those from Australia, Canada, and Europe. Given the prevalent infection patterns shared between cabbages in Europe and oilseed rape [32], the efficacy of ‘the Korean Differential Set’ may extend beyond Asia. However, its effectiveness might be limited to regions with extensive Chinese cabbage cultivation.

### 1.7. Chinese Sinitic Clubroot Differential Set

As more clubroot-resistance genes are being identified in *B. rapa*, several commercial clubroot-resistant hybrids of Chinese cabbage have been used for local pathogen classification in China, Korea, and Japan [19,39]. However, the availability of commercial F1 hybrids of Chinese cabbage suitable for clubroot differentiation is only sometimes assured, posing a potential limitation to their future utility [46]. Moreover, specific clubroot-resistant genes exhibit complete dominance in inheritance [48,49], while others follow a quantitative inheritance pattern [50]. In such instances, using F1 hybrids as hosts for differentiating pathotypes of *P. brassicae* is not recommended. Additionally, the genetic background and clubroot-resistant genes often need to be clarified for most hosts used in clubroot differentiation sets, with exceptions such as hosts ECD01 to ECD04 [51]. Hence, Pang et al. [37] have developed a set of differential hosts known as the Sinitic Clubroot Differential (SCD) set. This set utilizes eight clubroot-resistant inbred lines of Chinese cabbage with known resistance gene(s), along with a clubroot susceptible inbred line of Chinese cabbage, BJN3-1, to provide a stable platform for characterizing *P. brassicae* pathotypes (Table 7).

The populations of *P. brassicae* were inoculated onto each host within the SCD set and disease development was assessed six weeks post-inoculation. Resistance was determined if the mean disease index was below 25 and its associated 95% confidence interval did not overlap 50%; otherwise, susceptibility was concluded. In theory, this system has the capability to distinguish 256 pathotypes of *P. brassicae*. However, Pang et al. [37] identified 16 distinct pathotypes among 132 field isolates.

The advantageous aspect of the SCD set is the presence of known resistance genes, offering a significant opportunity to develop differential host sets for pathogen differentiation and the breeding of resistant cultivars.

## 2. Limitations of the Current Host Differential Sets

All existing systems exhibit both advantages as well as limitations in identifying the pathotypes from each Brassica species and characterizing their virulence spectrum or pattern on Brassica genotypes. Table 8 presents a summary of the comparison of all classification systems listed in this review. While variations exist in the dates of inoculation, types and quantities of inoculum, and disease assessment data, determining an appropriate threshold for resistance and susceptibility of genotypes remains a challenging task.

Moreover, the increase in *P. brassicae* populations capable of overcoming the resistance of Brassica cultivars presents an additional challenge in classifying pathotypes using current classification systems. The overcoming of clubroot-resistant cultivars may arise from the selective propagation of pathogenic genotypes on the Brassica cultivars. Each field population of *P. brassicae* is often heterogeneous and comprises multiple pathotypes, as evidenced by analyses of the pathogenicity of single-spore isolates of *P. brassicae* obtained from a single gall or field population [8,43,53,54,55]. Therefore, the optimal assessment of pathotypes should begin with isolating single spores to ensure that the study relies on pure genetic variants rather than mixtures of pathotypes. This effort is significantly challenging due to the potential presence of mixed strains within most *P. brassicae* populations found in individual root galls, as documented in canola roots in Alberta [56]. In this investigation, RNase H-dependent PCR (rhPCR) analyses revealed that 50 out of 79 galls contained *P. brassicae* populations including more than one strain. Consequently, researchers should aim to identify the prevalence of single spore isolates in the field and evaluate their relative proportions. The discovery that over 60% of galls contain ‘more than one’ isolate presents a formidable challenge, given the spatial and temporal limitations natural in pathotype classifications.

Moreover, international research teams should attempt to utilize assessments using near-isogenic lines with single resistance (R) genes, aiming to approach the detection of gene-for-gene relationships as closely as possible. This effort may prove challenging given the complexity of the *Brassica-Plasmodiophora* pathosystem, which encompasses numerous genes, gene variants, and types, along with a multitude of gene combinations. Additionally, discrepancies in the nomenclature of the same genes among various research teams and the identification of duplicates of the same gene [57] further complicate matters, potentially leading to significant variations in plant reactions.

Navigating towards standardized pathotyping tests should also involve considerations regarding the severity scales used, uniform formulas for calculating disease indices, and consistent thresholds. Broader scales, such as the 0–4 scale used by Kim et al. [39], straightforward calculations like the formula proposed by Horiuchi and Hori [58], which should be modified to the scale 0–4, and stringent cutoff thresholds e.g., 25%, as suggested by Somé et al. [35] appear to be the most suitable choices. 

Recent study suggested that the evolution within a given pathotype is driven by changes in the aggressiveness of the pathogen [59]. The further intensive cultivation of oilseed rape and the popularity of cruciferous vegetables may accelerate the emergence of new pathotypes. The pathotype designation system will therefore also have to undergo evolutionary changes.

## 3. Concluding Remarks

Balancing regional or national priorities while simultaneously harmonizing or standardizing international systems presents a significant challenge. The designation of ’custom-made’ pathotypes modified for nationwide screening or breeding purposes by specific companies offers numerous advantages. Users can precisely track the development of new pathotypes or races and compare current and previous pathogen populations, allowing conclusions to be drawn regarding the direction of their evolution. However, interpreting data internationally becomes challenging, if possible. The addition or deletion of a host differential, even among many, alters the pathotype designation. Frequently, new pathotyping systems utilize only a subset of existing differential sets and introduce their own new hosts, leading to considerable confusion. Nevertheless, the unique composition, diversity, and continual changes in local pathogen populations often necessitate such an approach. Furthermore, some users primarily focus on a specific Brassica host (e.g., *B. napus* or *B. rapa*), representing the unjustified use of host differentials from other Brassica species.

Using plant host differentials that remain consistently susceptible or resistant to the studied isolates not only expands the screening area unnecessarily but also entails excessive work and costs without yielding any novel or conclusive results. Consequently, adopting the ‘Global Host Differential Set’ by breeders seems improbable despite the potential benefits it could offer for population studies of *P. brassicae*.

Due to the increasing cultivation of winter oilseed rape, spring canola, and the popularity of vegetable brassicas combined with short crop rotations, we are witnessing a rapid emergence of new pathotypes of *P. brassicae*. Furthermore, some pathogen populations, once confined to small areas, have now significantly expanded. Given the extensive breeding efforts to develop clubroot-resistant cultivars, numerous independent differential host series will likely continue to be utilized, with new ones being developed over time.

The promising prospect lies in genetic tools currently being used to sequence the entire genomes of the pathogen. This advancement is expected to facilitate comparisons between isolates of *P. brassicae* and pathotypes from various locations. However, genetic descriptions must be accompanied by screening test results using as extensive of a differential set as possible. It is also essential to use the seeds of host differentials from safe and reliable seed suppliers. Essentially, by navigating toward race-specific phenotyping and utilizing single spore isolates and near-isogenic lines that vary with R genes, we can significantly advance research on the current composition and shifts in *P. brassicae* populations. Harmonizing evaluation systems through the adoption of consistent symptom evaluation scales, disease index calculation formulas, and disease thresholds, as well as striving to match the Global Host Differential Set as closely as possible, are important steps in this advancement. Both basic and applied research stand to gain substantial benefits from this level of standardization.

## Figures and Tables

**Table 1:** *Plasmodiophora brassicae*-pathotype classification scheme on the hosts of the American Differential set proposed by Williams [33].

Differential Host	Resistance Genes	Pathotype Designation															
		1	2	3	4	5	6	7	8	9	10	11	12	13	14	15	16
*Brassica oleracea*																	
cv. Jersey Queen	n. i.	+	+	+	+	−	+	+	−	−	+	−	+	−	−	−	−
cv. Badger shipper	n. i.	**−**	+	−	+	−	−	+	−	−	+	+	−	+	+	+	−
*Brassica napobrassica*																	
cv. Laurentian	n. i.	+	+	+	+	−	−	−	+	+	−	+	−	+	−	−	−
cv. Wilhelmsburger	n. i.	+	−	−	+	−	−	−	−	+	+	+	+	−	+	−	+

n. i.: not identified; + indicates a susceptible host reaction; − indicates a resistant host reaction.

**Table 2:** European Clubroot Differential (ECD) set with associated host numbers and binary and denary values developed by Buczacki et al. [34] ^a^.

Differential Number	Host	Binary Number	Denary Number
20-chromosome group (*Brassica rapa*)			
01	var. *rapifera* line aaBBCC	2^0^	1
02	var. *rapifera* line AAbbCC	2^1^	2
03	var. *rapifera* line AABBcc	2^2^	4
04	var. *rapifera* line AABBCC	2^3^	8
05	var. *pekinensis* cv. Granaat	2^4^	16
38-chromosome group (*Brassica napus* var. *napus*)			
06	line Dc101–cv. Nevin	2^0^	1
07	line Dc119–cv. Giant Rape	2^1^	2
08	line Dc128–cv. Giant Rape selection	2^2^	4
09	line Dc129–cv. New Zealand	2^3^	8
10	line Dc130–cv. Wilhemsburger	2^4^	16
18-chromosome group (*Brassica oleracea*)			
11	var. *capitata* cv. Badger Shipper	2^0^	1
12	var. *capitata* cv. Bindsachsener	2^1^	2
13	var. *capitata* cv. Jersey Queen	2^2^	4
14	var. *capitata* cv. Septa	2^3^	8
15	var. *fimbriata* cv. Verheul	2^4^	16

^a^ In this system, the differential hosts are arranged in a fixed order and each is assigned a denary number, 1, 2, 4, 8, 16, etc.; these numbers correspond to the binary series 2^0^, 2^1^, 2^2^, 2^3^, and 2^4^.

**Table 3:** *Plasmodiophora brassicae*-pathotype classification scheme on French Differential Set, proposed by Somé et al. [35].

Differential Host	Resistance Genes	Pathotype Designation							
		P1	P2	P3	P4	P5	P6	P7	P8
ECD 06	n. i.	+	+	−	−	−	+	−	+
ECD 10	n. i.	+	−	−	−	+	−	+	+
cv. Brutor	n. i.	+	+	+	−	+	−	−	−

ECD 06: *Brassica napus* var. *napus* cv. Nevin; ECD 10: *B. napus* var. *napus* cv. Wilhemsburger; cv.^.^ Brutor: spring oilseed rape *B. napus.* n. i.: not identified; (+) indicates a susceptible host reaction for a cut-off point of 25%; (−) indicates a resistant host reaction for a cut-off point of 25%.

**Table 4:** *Plasmodiophora brassicae*-pathotype classification scheme on the *Brassica rapa* hosts of the Japanese Clubroot Differential Set proposed by Osaki et al. [45].

Differential Host	Resistance Genes	Pathotype Designation			
		A	B	C	D
CR Kanki 100	n. i.	+	+	−	−
CR Kanko	n. i.	+	+	−	−
Utage 70	n. i.	+	−	+	−
Kukai 65	n. i.	+	+	−	−
Ryutoku	n. i.	+	+	−	−
Fukutakara 70	n. i.	+	+	−	−
Nozaki Nigo ^a^	none	+	+	+	+

^a^ Susceptible *B. rapa* cultivar used as control; n. i.: not identified; (+) compatible; (−) incompatible.

**Table 5:** *Plasmodiophora brassicae*-pathotype classification scheme on the hosts of the Canadian Clubroot Differential (CCD) set by Strelkov et al. [36].

					Pathotype Designation ^a^												
CCD	A	B	C	D	E	F	G	H	I	J	K	L	M	N	O	P	X
Williams	3	2	5	3	8	2	5	3	5	8	5	5	6	8	3	8	5
Somé	P2	P2	P2	P2	P2	P2	P3	P2	P2	P3	P3	P3	P2	P2	P3	P2	P3
**Differential Host**								**Reaction ^b^**									
ECD 02	−	−	−	−	−	−	−	−	−	−	−	−	−	−	−	−	−
ECD 05	+	+	+	+	+	+	+	+	+	+	+	+	+	+	+	+	+
ECD 06	+	+	+	+	+	+	−	+	+	−	−	−	+	+	−	+	−
ECD 08	+	+	+	+	+	+	+	+	+	+	−	+	+	+	+	+	+
ECD 09	+	+	+	+	+	+	−	+	+	−	−	−	+	+	+	+	−
ECD 10	−	−	−	−	−	−	−	−	−	−	−	−	−	−	−	−	−
ECD 11	−	+	−	−	−	+	−	−	−	−	−	−	−	−	−	−	−
ECD 13	+	+	−	+	−	+	−	+	−	−	−	−	+	−	−	−	−
Brutor	+	+	+	+	+	+	+	+	+	+	+	+	+	+	+	+	+
Laurentian	+	+	−	+	+	+	−	+	−	+	−	−	−	+	+	+	−
Mendel	+	+	−	−	−	−	−	−	−	−	−	−	−	−	−	+	+
Westar	+	+	+	+	+	+	+	+	+	+	+	+	+	+	+	+	+
45H29	+	+	+	+	+	−	+	−	−	+	+	−	−	−	+	+	+

^a^ CCD pathotypes F, H, I, M, and N correspond to the original pathotypes 2, 3, 5, 6, and 8, as defined on the differentials of Williams [33], which cannot overcome resistance in any clubroot resistant *B. napus* host; CCD pathotype L corresponds to the *P. brassicae* population D-G3 reported by Strelkov et al. [27], while CCD pathotype X corresponds to the populations L-G1, L-G2, and L-G3 reported by Strelkov et al. [27]. ECD 02: *Brassica rapa* var. *rapifera* line aaBBCC: ECD 05: *B. rapa* var. *pekinensis* cv. Granaat; ECD 06: *B. napus* var. *napus* cv. Nevin; ECD 08: *B. napus* var. *napus* cv. Giant Rape selection; ECD 09: *B. napus* var. *napus* cv. New Zealand; ECD 10: *B. napus* var. *napus* cv. Wilhemsburger; cv. Brutor: spring oilseed rape *B. napus;* Laurentian: *B. napobrassica cv.* Laurentian; Mendel: first clubroot-resistant *B. napus* cultivar; Westar: the open-pollinated canola; 45H29: clubroot-resistant hybrid canola. ^b^ plus (+) sign denotes a susceptible host reaction, while a minus (−) sign denotes a resistant reaction. Host reactions were classified as + or − based on the index of disease (ID) that developed 6 weeks following inoculation; for a reaction to be considered resistant, the mean ID must be <50% and the 95% confidence interval must not exceed 50%.

**Table 6:** *Plasmodiophora brassicae*-pathotype classification scheme on the Korean clubroot differential set developed by Kim et al. [39].

Cultivar	Pathotype Designation			
	Pathotype 1	Pathotype 2	Pathotype 3	Pathotype 4
Noranggimjang	S	S	S	S
CR-Cheongrok	R	R	S	S
DegaoCR1016	R	S	R	S
Akimeki	R	R	R	S

S: susceptible response; R: resistant response.

**Table 7:** *Plasmodiophora brassicae*-pathotype classification scheme on the *Brassica rapa* hosts of the Chinese Sinitic Clubroot Differential Set presented by Pang et al. [37].

Differential Host	Resistance Genes	Pathotype Designation															
		1	2	3	4	5	6	7	8	9	10	11	12	13	14	15	16
CR-096	Novel CR gene(s) ^a^	−	−	−	−	−	−	−	−	−	+	−	−	−	−	−	−
CR-20	*Crr1* ^a^, *Crr4* ^a^	−	−	−	−	−	−	−	−	+	+	+	−	−	+	+	+
CR-26	*Crr1* ^a^	−	−	−	−	−	−	+	+	+	+	+	−	−	−	+	−
CR-77	*CRa* ^b^, *Crr1* ^a^	−	−	−	−	−	+	−	+	+	+	+	−	+	−	+	+
222	*CRa* ^c^	−	−	−	+	+	+	−	+	−	−	+	−	−	+	+	+
CR-75	*CRa* ^b^, *Crr2* ^c^	−	−	+	+	−	−	−	−	−	−	+	+	−	+	−	+
85–74	*CRd* ^b^	−	−	−	−	−	−	−	−	−	−	−	−	+	−	−	−
CR-73	*Crr3* ^b^, *Crr4* ^b^	−	+	−	−	−	−	−	−	−	−	−	−	−	−	−	−
BJN3-1	None	+	+	+	+	+	+	+	+	+	+	+	+	+	+	+	+

^a^ Landraces; ^b^ Pang et al. [49]; ^c^ Chen et al. [52]; (+) sign denotes a susceptible host reaction, while a minus (−) sign denotes a resistant reaction. Host reactions were classified as + or − based on the disease index (DI) that developed 6 weeks following inoculation. The resistance was determined if the mean DI was lower than 25 and its associated 95% confidence interval did not overlap 50%.

**Table 8:** Summary of the comparison of all classification systems for the categorization of *Plasmodiophora brassicae* populations.

Classification Systems	Number of Plant Species	*Brassica* Species	Plant Age at the Time of Inoculation (Days)	Inoculum Type	Inoculum Concentration	Disease Assessment Date	Scale	Disease Index (DI)	Cut-Off Point
American set ^a^	4	2 × *B. napobrassica* 2 × *B. oleracea*	7	infested soil	1 × 10^8^ spores/cc soil	35 dpi ^i^	0–3	yes	not defined
European Clubroot Differentials ^b^	15	5 × *B. rapa* 5 × *B. napus* 5 × *B. oleracea*	10	spore suspension	1 × 10^7^ spores/mL	35–56 dpi	0–3	yes	33%
French set ^c^	3	3 × *B. napus*	6 to 10	spore suspension	1 × 10^4^ spores/mL	35–42 dpi	0–3	yes	25%
Japanese set ^d^	6	6 × *B. rapa*	0 ^h^	infested soil	5 × 10^6^ per g soil	42 das ^j^	0–3	no	DS ≤ 1
Korean set ^e^	4	4 × *B. rapa*	10	spore suspension	5 × 10^7^ spores/mL	30 dpi	0–4	no	DS ≤ 1
Canadian Clubroot Differentials ^f^	13	2 × *B. rapa* 9 × *B. napus* 2 × *B. oleracea*	6	spore suspension	1 × 10^7^ spores/mL	42 dpi	0–3	yes	50%
Chinese Sinitic Clubroot Differentials ^g^	8	8 × *B. rapa*	5	spore suspension	1 × 10^7^ spores/mL	42 dpi	0–3	yes	25%

^a^ Williams [33]; ^b^ Buczacki et al. [34]; ^c^ Somé et al. [35]; ^d^ Osaki et al. [45]; ^e^ Kim et al. [39]; ^f^ Strelkov et al. [36]; ^g^ Pang et al. [37]; ^h^ seed sown directly into infested soil; ^i^ days post inoculation (dpi); ^j^ days after sowing (das).

## Data Availability

Not applicable.
